# Evaluation of Multiple Immunoassay Technology Platforms to Select the Anti-Drug Antibody Assay Exhibiting the Most Appropriate Drug and Target Tolerance

**DOI:** 10.1155/2016/5069678

**Published:** 2016-05-03

**Authors:** Justine Collet-Brose, Pierre-Jean Couble, Maureen R. Deehan, Robert J. Nelson, Walter G. Ferlin, Sabrina Lory

**Affiliations:** Novimmune SA, 14 Chemin des Aulx, 1228 Plan-les-Ouates, Geneva, Switzerland

## Abstract

The aim of this study was, at the assay development stage and thus with an appropriate degree of rigor, to select the most appropriate technology platform and sample pretreatment procedure for a clinical ADA assay. Thus, ELISA, MSD, Gyrolab, and AlphaLISA immunoassay platforms were evaluated in association with target depletion and acid dissociation sample pretreatment steps. An acid dissociation step successfully improved the drug tolerance for all 4 technology platforms and the required drug tolerance was achieved with the Gyrolab and MSD platforms. The target tolerance was shown to be better for the ELISA format, where an acid dissociation treatment step alone was sufficient to achieve the desired target tolerance. However, inclusion of a target depletion step in conjunction with the acid treatment raised the target tolerance to the desired level for all of the technologies. A higher sensitivity was observed for the MSD and Gyrolab assays and the ELISA, MSD, and Gyrolab all displayed acceptable interdonor variability. This study highlights the usefulness of evaluating the performance of different assay platforms at an early stage in the assay development process to aid in the selection of the best fit-for-purpose technology platform and sample pretreatment steps.

## 1. Introduction

Monoclonal antibodies have been successfully used as therapeutic agents for the treatment of diseases including breast cancer, leukemia, asthma, arthritis, psoriasis, Crohn's disease, and transplant rejection [1–5]. As part of a therapeutic antibody clinical development program it is necessary to evaluate the immunogenic potential of the antibody. This is measured as an anti-drug antibody (ADA) response and if it occurs it can cause undesired effects ranging from loss of drug exposure and loss of efficacy to serious adverse events. Therefore, immunogenicity assessment is a regulatory requirement of clinical studies [6–10].

Testing of ADA to therapeutic proteins is typically performed using a tiered approach [7–12]. The samples are initially screened for their ability to bind the therapeutic drug, screened positive samples are then confirmed in a second assay, and their isotype and neutralizing capacity can also be evaluated. In recent times, the most common ADA assay format is a bridging assay and the traditional enzyme-linked immunosorbent assay (ELISA) is often a suitable option. However, new immunoassay platforms have been developed including MSD, Gyrolab, and AlphaLISA with improved sensitivity, accuracy, variability, reduced assay time, and reduced sample volume requirements.

**Figure 1:**
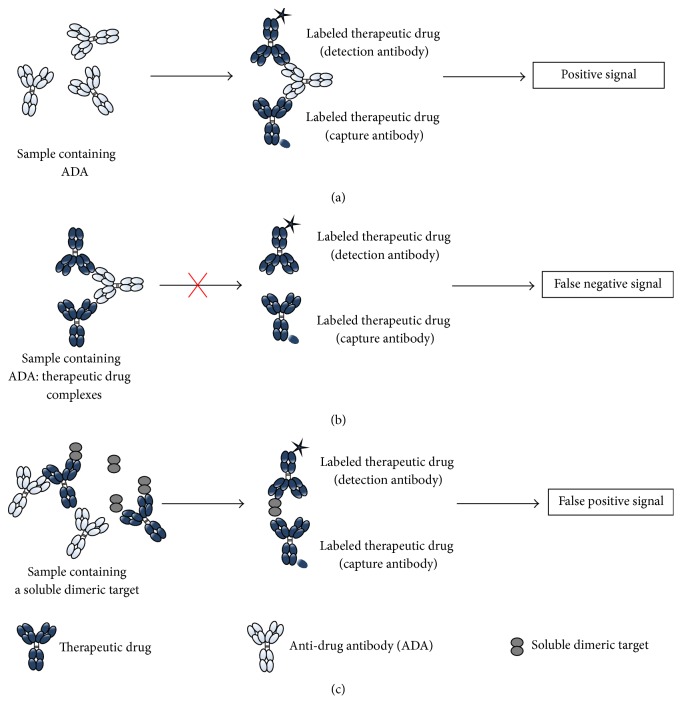
Schematic representation of different scenarios that can occur in the bridging assay. (a) Presence of ADA leading to a positive signal. (b) Drug interference leading to false negative signal. (c) Target interference leading to false positive signal.

Figure 1(a) is a schematic representation of a typical bridging assay format where the ADA bridges two molecules of therapeutic drug labeled with different tags and elicits a signal (e.g., fluorescence and electrochemiluminescence) that can be measured by a reader. With this assay format, the most common form of interference is from the therapeutic protein itself. Therapeutic drug in the clinical sample is able to bind ADA and prevents it from forming a complex with the capture and detection reagents and thus can lead to a false negative result during the clinical sample analysis (Figure 1(b)). The ability of the assay to detect ADA in the presence of therapeutic drug is called drug tolerance and this parameter needs to be addressed during assay development. Drug interference is common in preclinical toxicology studies and in multiple dose clinical trials where high therapeutic drug concentrations are reached. Drug interference can be mitigated by collecting samples for ADA testing at late time points in the clinical trial when the concentration of the therapeutic is expected to be lower, for example, after the wash-out period or at the end of the clinical trial. However, in order to make ADA assessments at appropriate time points in relation to the underlying disease, drug interference often has to be addressed methodologically. Over the last few years, the challenge of drug interference in ADA assays has often been overcome by performing an acid dissociation of the therapeutic drug-ADA complex step, followed by a neutralization step in the presence of the capture and detection reagents [13–18].

A second challenge observed during immunogenicity assessment with a bridging assay format is interference due to the target. The presence of dimeric or multimeric forms of soluble target in a clinical sample may lead to the bridging of the capture and detection reagent and can lead to a false positive result during the clinical sample analysis (Figure 1(c)). Pretreatment with blocking antibodies to the target, blocking target-binding proteins, and immunodepletion of the target can eliminate this type of interference [19].

In our study, we describe the development of an ADA assay for a therapeutic antibody directed against a soluble dimeric target (Novimab) with evaluation of different technology platforms (ELISA, MSD, Gyrolab, and AlphaLISA) and different additional steps (±acid dissociation, ±target depletion) in order to achieve an assay with an appropriate sensitivity, drug tolerance, and target tolerance for our clinical trial population. Appropriate sensitivity was defined as 50 ng/mL reference ADA, somewhat more sensitive than the 250–500 ng/mL level suggested in industry guidelines. As the sensitivity parameter was defined using a polyclonal reference ADA purified from immunized rabbit serum, likely to differ in affinity, avidity, and antigenic specificity to a real ADA response in treated subjects, it was felt that an assay with greater sensitivity would be more appropriate to detect low level immunogenicity events. Appropriate drug tolerance and target tolerance were defined as 20 *μ*g/mL Novimab and 50 ng/mL target, respectively, levels defined with the clinical pharmacologist based on PK-PD modeling of the likely accumulation of therapeutic drug and target in the clinical population with the proposed study dosing regimen. Sensitivity, interdonor variability, drug tolerance, and target tolerance were used as the key parameters to assess during this assay development and technology evaluation study. The goal of this work was not to validate an assay on each platform but to evaluate the critical parameters on each platform in order to select the most appropriate assay for subsequent validation and clinical sample analysis.

## 2. Materials and Methods

### 2.1. Reagents, Assay Materials, and Serum

The fully human IgG1 therapeutic monoclonal antibody (Novimab) was produced at Novimmune. The anti-drug polyclonal antibody (reference ADA) purified from immunized rabbit serum was supplied by Agro-Bio (La Ferté-Saint-Aubin, France). The soluble dimeric target was purchased from R&D Systems (Minneapolis, MN, United States). The biotinylated nonblocking anti-target mouse monoclonal antibody was purchased from Mabtech (Stockholm, Sweden). Streptavidin magnetic beads were supplied by Thermo Fisher Scientific (Waltham, MA, United States). Horseradish Peroxidase- (HRP-) conjugated mouse-anti-FITC IgG Fraction Monoclonal was purchased from Jackson Immunoresearch (West Grove, PA, United States). 30 individual human sera and a pooled human serum were purchased from SeraLab (West Sussex, United Kingdom). Tetramethylbenzidine (TMB) substrate and the Stop Solution for TMB substrate were supplied by Sigma-Aldrich (Postfach, Switzerland). 96-well round bottomed polypropylene plates and 96-skirt PCR plates were purchased from Fisher Scientific (Wohlen, Switzerland). 96-well streptavidin plates were purchased from Roche Diagnostics (Basel, Switzerland). High binding streptavidin plates and the 4x MSD read buffer were supplied by Meso Scale Discovery (Rockville, MD, United States). Gyrolab Bioaffy 200 CDs, Gyrolab ADA CDs, Rexxip F, and Rexxip ADA were purchased from Gyros AB (Uppsala, Sweden). 96-well 1/2 area white plates, acceptor beads, and donor beads were purchased from PerkinElmer (Lausanne, Switzerland). SULFO-TAG-, FITC-, and acceptor beads-labeled Novimab were prepared at Novimmune according to the recommendations from Meso Scale Discovery (Rockville, MD, United States), Thermo Fisher Scientific (Waltham, MA, United States), and PerkinElmer (Lausanne, Switzerland), respectively. Biotinylated- and Alexa Fluor 647-labeled Novimab were prepared by R&D Biotech (Besançon, France).

### 2.2. Equipment

Different readers were required for the different assay formats as follows.

#### 2.2.1. ELISA

Absorbance was measured on the Synergy HT instrument (Biotek, Luzern, Switzerland).

#### 2.2.2. MSD

Electrochemiluminescence was measured on the Sector Imager 6000 instrument (Meso Scale Discovery, Rockville, MD, United States).

#### 2.2.3. Gyrolab

Fluorescence was measured on the Gyrolab Workstation with the Gyrolab Control software (v5.4.0) (Gyros AB, Uppsala, Sweden).

#### 2.2.4. AlphaLISA

Fluorescence was measured on the Synergy NEO instrument (Biotek, Luzern, Switzerland).

### 2.3. Sample Preparation

All samples were prepared on the same day, aliquoted into 96-well polypropylene plates, and stored at −80°C.

#### 2.3.1. Standard Samples

A reference ADA standard (from 0.34 to 20 ng/mL) was prepared in neat pooled human serum. The negative control (NC) sample consisted of neat pooled human serum alone. For the comparison of the different platforms, relative quantitation against this reference ADA was performed. In the final assay format, a tiered approach was used with the reference ADA being used to evaluate system suitability.

#### 2.3.2. PC and Drug Interference Samples

Positive Control (PC) samples were prepared to final concentrations of 2, 5, 20, 50, 100, 250, 500, 1000, and 10000 ng/mL, by diluting the reference ADA in pooled human serum. Novimab samples were prepared to final concentrations of 0, 20, and 100 *μ*g/mL in pooled human serum. Samples were incubated at 22°C for 1 h with agitation (450 rpm) to allow Novimab: ADA complex formation.

#### 2.3.3. Target Interference Samples

Target interference samples were prepared by diluting the soluble dimeric target to final concentrations of 5, 20, 50, and 200 ng/mL in pooled human serum.

#### 2.3.4. Selectivity

Thirty individual human sera and one pooled human serum were tested. The pooled human serum was also used for the preparation of the standard, NC, PC, drug interference, and target interference samples.

### 2.4. Sample Pretreatment

#### 2.4.1. Target Depletion

For each technology evaluated, the samples spiked with the soluble dimeric target were tested under two conditions: nondepleted or depleted. The nondepleted samples were diluted at 1 : 3 in PBS-1% BSA 0.05% tween. The depleted samples were first diluted at 1 : 2 with a biotinylated noncompetitive anti-target mouse monoclonal antibody and incubated at 22°C for 1 h with agitation (450 rpm). Two volumes of this solution were then mixed with one volume of prewashed streptavidin magnetic beads. After 1 h incubation at 22°C with agitation (450 rpm) the plate was placed on a magnet for 2 minutes. The supernatant was then removed with a pipette and used in the subsequent steps of the assay.

#### 2.4.2. Acid Treatment

For each technology evaluated, the samples were tested under two conditions: nonacidified or acidified. No pretreatment was applied to the nonacidified samples. The acid dissociation method was optimized on the ELISA platform by testing various parameters. Several acid solutions were compared including 100 mM, 300 mM, and 600 mM acetic acid, 50 mM and 100 mM hydrochloric acid, and 0.5 M Glycine at pH 2.2 and 2.6. Sample : acid ratios at 1 : 2, 1 : 3, 1 : 5, and 1 : 10 (volume : volume) were tested and acidification time of 20 and 40 minutes was compared. Similarly the neutralization step was optimized by preparing Tris solutions at different molarities (from 0.1 to 2 M), at different pH (from 7.5 to 9.0) and neutralization time of 2 hours and 16 hours for comparison. All of these optimizations were conducted on samples containing reference ADA concentrations ranging from 0 to 10000 ng/mL complexed with 0 or 20 *μ*g/mL of Novimab. Based on these experiments 1 in 5 dilutions of sample in 300 mM acetic acid was selected as the most suitable, combining appropriate sensitivity, drug tolerance, and sample usage. The suitability of this sample acidification procedure was then confirmed on the other plate-based platforms (i.e., MSD and AlphaLISA). For the Gyrolab platform, a specific optimization of the acid treatment was performed due to the use of the Gyrolab Mixing CDs, where a sample : acid ratio of 1 : 1 (volume : volume) was used due to the volume definition of the CD microstructures. The samples that required an acidification were treated as follows.

*(i) ELISA, MSD, and AlphaLISA*. 20 *μ*L of each sample was mixed with 80 *μ*L of 300 mM acetic acid in a 96-well round-bottom polypropylene plate. The acidified samples were then incubated at 22°C for 40 minutes with agitation (450 rpm).

*(ii) Gyrolab*. A Gyrolab Mixing CD was used in order to have a fully automated acid treatment step. 200 nL of sample was transferred into the reacting chamber of the Mixing CD and mixed with 200 nL of 0.5 M Glycine-HCl pH 2.6. This was then incubated for 10 minutes at room temperature.

### 2.5. ADA Screening Assay

#### 2.5.1. ELISA

*(i) For the Nonacidified Samples*. 2x Master Mix containing 2 *μ*g/mL biotin- and 2 *μ*g/mL FITC-labeled drug was prepared in PBS-1% BSA 0.05% tween. One volume of Master Mix was added to one volume of sample and incubated for 16–24 h at 22°C, with agitation (450 rpm).

*(ii) For the Acidified Samples*. 3x Master Mix containing 3 *μ*g/mL biotin- and 3 *μ*g/mL FITC-labeled drug was prepared in PBS-1% BSA 0.05% tween. One volume of Master Mix, one volume of acidified samples, and one volume of 1 M Tris pH 8.0 were mixed together and incubated for 2 h at 22°C, with agitation (450 rpm).

*(iii) For Both Treatments*. A 96-well streptavidin plate was blocked with 200 *μ*L/well of PBS-3% BSA for 1 to 4 h and washed three times with PBS-0.05% tween. The sample/conjugate mix (45 *μ*L/well) was pipetted into the blocked streptavidin plate and incubated at 22°C for 1 h with agitation (450 rpm). The plate was washed three times using PBS-0.05% tween followed by the addition of 50 *μ*L/well of 1/8000 diluted HRP-conjugated mouse-anti-FITC IgG Fraction Monoclonal. The plate was incubated at 22°C for 1 h with agitation (450 rpm) and then washed three times with PBS-0.05% tween. The TMB substrate was added to each well (50 *μ*L/well) and incubated at 22°C for 5 minutes. The reaction was stopped by the addition of 50 *μ*L/well of Stop Solution for TMB substrate and absorbance was measured at 450 nm on the Synergy plate reader.

#### 2.5.2. MSD

*(i) For the Nonacidified Samples*. 2x Master Mix containing 4 *μ*g/mL biotin- and 4 *μ*g/mL SULFO-TAG-labeled drug was prepared in PBS-1% BSA 0.05% tween. One volume of Master Mix was added to one volume of sample and incubated for 16–24 h at 22°C, with agitation (450 rpm).

*(ii) For the Acidified Samples*. 3x Master Mix containing 6 *μ*g/mL biotin- and 6 *μ*g/mL SULFO-TAG-labeled drug was prepared in PBS-1% BSA 0.05% tween. One volume of Master Mix, one volume of acidified samples, and one volume of 1 M Tris pH 8.0 were mixed together and incubated for 2 h at 22°C, with agitation (450 rpm).

*(iii) For Both Treatments*. A MSD high binding avidin plate was blocked with 200 *μ*L/well of PBS-3% BSA for 1 to 4 h and washed three times with PBS-0.05% tween. The sample/conjugate mix (45 *μ*L/well) was pipetted into the blocked plate and incubated at 22°C for 1 h, with agitation (450 rpm). The plate was then washed three times using PBS-0.05% tween and 150 *μ*L of the 2x MSD Read Buffer was transferred into each well. The electrochemiluminescence signal was then measured on the Sector Imager 6000 instrument.

#### 2.5.3. AlphaLISA

*(i) For the Nonacidified Samples*. 2.5x Master Mix containing 5 nM biotin-labeled drug and 50 *μ*g/mL drug coated acceptor beads was prepared in PBS-1% BSA 0.05% tween. Two volumes of Master Mix were added to one volume of sample and incubated for 16–24 h at 22°C, with agitation (450 rpm). Streptavidin donor beads were prepared at 50 *μ*g/mL in PBS-1% BSA 0.05% tween and diluted at 1 : 2.5 with the sample/acceptor beads mix. This solution was incubated at 22°C for 1 h, with agitation (450 rpm).

*(ii) For the Acidified Samples*. 6x Master Mix containing 12 nM biotin-labeled drug and 120 *μ*g/mL drug coated acceptor beads was prepared in PBS-1% BSA 0.05% tween. One volume of Master Mix, two volumes of acidified samples, and one volume of 1 M Tris pH 8.0 were mixed together and incubated for 16 to 24 h at 22°C, with agitation (450 rpm). Streptavidin donor beads were prepared at 60 *μ*g/mL in PBS-1% BSA 0.05% tween and diluted at 1 : 3 with the sample/acceptor beads mix. This solution was incubated at 22°C for 1 h with agitation (450 rpm).

*(iii) For Both Treatments*. 50 *μ*L of the sample/beads mix was transferred into a 96-well 1/2 area white plate and the plates were measured on the Synergy plate reader.

#### 2.5.4. Gyrolab

*(i) For the Nonacidified Samples*. 2x Master Mix containing 4 *μ*g/mL biotin- and 4 *μ*g/mL Alexa Fluor 647-labeled drug was prepared in Rexxip F. One volume of Master Mix was added to one volume of sample and incubated for 16–24 h at 22°C, with agitation (450 rpm) and transferred into a 96-well skirted PCR plate. The plate was loaded into the Gyrolab platform together with a Gyrolab Bioaffy 200 CD. The run was designed with the Gyrolab Client v5.4.0 software and the data were analyzed at the 5% photomultiplier tube setting using the Gyrolab Evaluator v3.3.7.171 software.

*(ii) For the Acidified Samples*. 3x Master Mix containing 6 *μ*g/mL biotin- and 6 *μ*g/mL Alexa Fluor 647-labeled drug was prepared in a 2 M Tris pH 8.0 : Rexxip ADA buffer (1 : 1 vol) solution. The samples, the Master Mix, and 0.5 M Glycine pH 2.6 solution were transferred into a 96-well skirted PCR plate. The plate was loaded into the Gyrolab platform together with a Gyrolab Mixing CD. The run was designed with the Gyrolab Client software in order to achieve an acidification and a neutralization time of 10 minutes each. The acidification was performed by mixing one volume of sample with one volume of 0.5 M Glycine pH 2.6 solution. One volume of Master Mix was then added to the acidified samples for the neutralization. The data were analyzed at the 5% photomultiplier tube setting using the Gyrolab Evaluator software.

### 2.6. Data Analysis

#### 2.6.1. Run Specific Cut-Point Determination

ADA assays require the determination of a cut-point to evaluate whether a sample is considered as positive (signal of the sample at or above the cut-point) or negative (signal of the sample below the cut-point). The calculation of this cut-point requires assessment of a large population of individual matrices and statistical analysis of the results. As the primary aim of this study was to evaluate and compare different technology platforms and respective methods, a statistical method using a floating cut-point was employed by calculating a run specific cut-point (RSCP) for each analysis plate/CD.

A limited number of naïve individual matrices (*n* = 30) were screened on each method. The outliers were then excluded using a BoxPlot analysis. The assay cut-point (ACP) was determined as follows:

(1) ACP=Mean  individual  matrix+1.645×Standard  deviation.

A normalization factor (NF) was calculated using the ACP and the NC from the 30 donor screening runs:

(2) NF=ACP−Mean  NC  of  the  runs  used  to  calculate  the  ACP.

Finally, the RSCP was calculated for each run using the run NC and the NF:

(3) $$\begin{array}{c} RSCP=\text{Mean NC of the run}+NF. \end{array}$$

#### 2.6.2. Threshold Determination

Due to issues encountered on the ELISA and the AlphaLISA platforms, the RSCP determination was found to be biased and of limited value for the technology evaluation. For the ELISA platform, signals very close to the lower detection limit of the reader (optical density below 0.05) were obtained for the individual donors leading to an underestimation of the interdonor variability. On the contrary, for the AlphaLISA technology, a high interdonor variability was observed. Whilst extended optimization work may have addressed these issues to some degree, for the comparison work performed as part of the assay development phase, these were viewed as limitations of the platforms. In addition, the evaluation performed with individual donors was limited during this development phase as compared to the comprehensive balanced design assessment that is typical of the assay validation phase, a further source of bias for the determination of the cut-point. For these reasons a statistical cut-point, from a limited individual population evaluation, was deemed inappropriate because it would not have permitted a fair comparison of the different methods.

Thus an arbitrary threshold (Th) was calculated, according to the following equation:

(4) $$\begin{array}{c} Th=\text{Mean NC of the run}\times 1.25. \end{array}$$

The value was set at 25% above the background, a level that would robustly distinguish a real change in signal response as a result of different experimental parameters to those that were due to method precision, thus multiplication factor of 1.25. Using the threshold method still allowed normalization for run-to-run variation and applied a consistent factor to all of the runs and across each of the different platforms. In this study, samples containing the same matrix (pool of sera) were used. Consequently, an approach based on the background of this matrix rather than on the interdonor variability was found to be more relevant.

### 2.7. Analysis Software and Parameters

All of the raw data generated on ELISA, MSD, and AlphaLISA were imported into the Gen5 analysis software (v1.6). The curves were generated using a 4-parameter analysis and weight 1/*y*. The raw data generated on Gyrolab were analyzed on the Gyrolab Evaluator software (v3.3.7.171) using the Quantification module (v3.3.7.171) and the ADA Analysis module (v3.3.7.171). The curves were generated using a 5-parameter analysis and weight 1/*y* ^2^.

### 2.8. Acceptance Criteria

The goal of this study was to select a fit-for-purpose method for further assay development. This study was conducted as a preliminary evaluation and acceptance criteria were set according to the individual project requirements.

#### 2.8.1. Sensitivity

Industry guidance recommends the development of an immunogenicity assay with a sensitivity of approximately 250–500 ng/mL [11, 12, 20]. However, the choice of the reference ADA used in the assay has a significant impact on the sensitivity achieved. Purified polyclonal rabbit anti-drug antibodies are commonly used in the development of immunogenicity assays at Novimmune and with this reference ADA it was felt that a lower sensitivity would be more appropriate in order to detect low level immunogenicity events. Consequently, the sensitivity acceptance criteria were set at 50 ng/mL in this study.

#### 2.8.2. Drug Tolerance

An ADA assay with an inappropriate drug tolerance leads to false negative samples. Based on PK-PD modeling of the likely drug accumulation with the clinical dosing regimen, the drug tolerance acceptance criteria were set at 20 *μ*g/mL in this study.

#### 2.8.3. Target Tolerance

In this ADA assay, lack of target tolerance would lead to the generation of false positive results. Based on the soluble dimeric target level in a disease population and its potential accumulation [21] during the treatment period, a target tolerance up to 50 ng/mL was considered as acceptable for this ADA assay.

#### 2.8.4. Interassay and Interdonor Variability

These two parameters are studied during an ADA assay validation. Consequently it was important to evaluate them in the assay development step to ensure they would not create any major issues during the assay validation phase. As a result, the data obtained from these parameters was only used for information but was not considered as critical as the drug and the target tolerance.

The interassay precision based on the PC signal was calculated over 3 runs and performed on 3 different days, for each method. The number of donors considered as positive (with a signal above the threshold) was analyzed to evaluate the interdonor variability. The method showing minimal interassay and interdonor variability would be selected.

## 3. Results and Discussion

**Figure 2:**
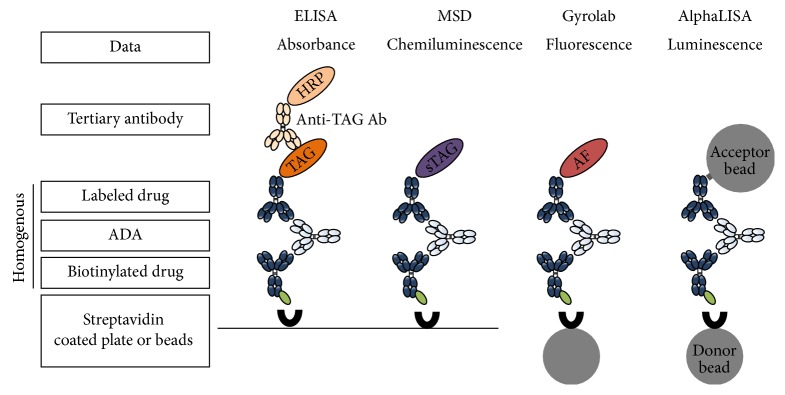
Assay formats and platforms. For each platform, a bridging format was used with the biotinylated drug (Novimab) as the capture antibody. The drug (Novimab) was also used as the detection antibody and was conjugated to a specific labeling molecule appropriate to each of the different platforms. Only the ELISA assay was developed using a tertiary antibody directed against the detection antibody TAG. HRP: Horseradish Peroxidase, sTAG: SULFO-TAG, and AF: Alexa Fluor 647.

The same bridging format immunogenicity assay using a labeled Novimab as a capture and detection reagent was developed and optimized in a homogenous manner on 4 different technology platforms: ELISA, MSD, Gyrolab, and AlphaLISA. The optimization included the determination of the capture/detection reagent concentrations, the minimum required dilution, and the incubation time. The different assay formats for the platforms are described in Figure 2.

**Figure 3:**
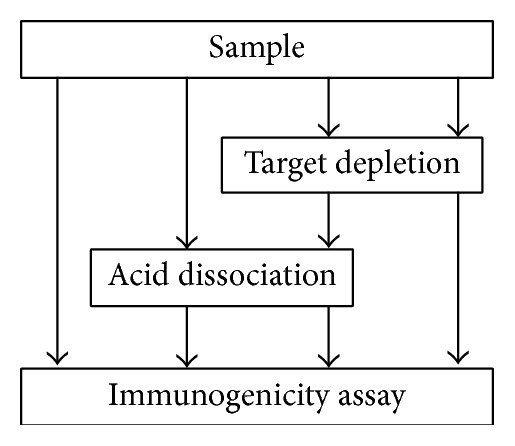
Samples (standard, NC, PC, drug, and target interference samples) were subjected to different treatments and analyzed in the ADA screening assay.

In the evaluation study, samples were subjected to different steps (±acid dissociation, ±target depletion), as shown in Figure 3, and analyzed on each of the 4 platforms. The objective was then to select the best fit-for-purpose method (combination of platform and additional steps). As elevated drug and target levels were expected in the clinical population, it was crucial to select a method able to tolerate these levels to expose true ADA positive events. The sensitivity of the assay was assessed to achieve a suitable level for the clinical sample testing phase and evaluate the concentration of the low PC concentration that would be used for system suitability purposes. The assessment of interassay and interdonor variability was also an important consideration.

During the optimization experiments of the bridging assay on the different platforms, different minimum required dilutions were obtained. Consequently, according to the pretreatment (additional step) and the platform used, the final dilutions applied to the samples varied and are summarized in Table 1. The samples submitted to a target depletion step were diluted at 1 in 3 due to the addition of the reagents used for the immunodepletion. To allow a direct comparison of the efficiency of the target depletion procedure, equivalent 1 in 3 dilutions in buffer was applied to samples not submitted to the target depletion step. For acid dissociation, samples were diluted at 1 in 5 with a low pH buffer for all technologies, except Gyrolab. For Gyrolab the volumes for the different sample and buffer addition steps are defined within the CD microstructure and fixed at 200 nL allowing only a sample : acid ratio of 1 : 1 (volume : volume). No additional dilution was applied to the samples requiring no acid dissociation treatment. The nonacidified samples were diluted at 1 in 2 with the Master Mix solution, except for the AlphaLISA platform where 1 in 5 dilutions was performed, as recommended by the provider. One in 3 dilutions was applied to the acidified samples as 1 volume of neutralization buffer had to be added in addition to the Master Mix solution. The Gyrolab technology was an exception as the Master Mix had to be prepared directly in the neutralization buffer and 1 volume of this solution added to 2 volumes of acidified sample.

### 3.1. Analysis Strategy

The goal of this evaluation was to select a fit-for-purpose method to support clinical development of Novimab. In the ADA assay, the determination of a cut-point is a key parameter because it defines the limit above which a result is considered as “positive” during the clinical sample testing phase. In this study, to allow evaluation of the different technologies, a relative-qualitative approach was taken, using a reference ADA to describe key assay attributes such as sensitivity, drug, and target tolerance and interdonor variability and interassay precision. Increasing concentrations of PCs were prepared and analyzed in the different methods to check whether the signal obtained was positive or negative (signal above or below the cut-point value, resp.). In the final method validation and sample analysis, the reference ADA would instead be used for system suitability assessment, ADA positivity of samples being evaluated following the tiered approach with semiquantitation by serial dilution titration.

Consequently, the cut-point parameter is critical and has to be carefully calculated. As explained in Section 2, a statistical approach based on a limited number of donors (30) was initially used to calculate the cut-point. Using this approach, a bias was observed for two methods, ELISA and AlphaLISA, without an acid dissociation step leading to a misinterpretation of the data. There are different reasons to explain this observation. For the ELISA platform, the signals given by the different donors were very close to the lower detection limit of the reader (optical density below 0.05), leading to an underestimation of the interdonor variability. On the contrary, for the AlphaLISA technology, a high interdonor variability was observed. Moreover, the number of donors used for the determination of the cut-point may have been too low to provide a robust value. For these reasons a statistical cut-point was deemed inappropriate because it would not have permitted a fair comparison of all of the methods. Furthermore, the determination of a robust cut-point requires the assessment of multiple donors (80 or more). This intensive approach is not normally required during the assay development phase.

Therefore, a second approach was used to set a limit above which a result is considered “positive.” For this approach, an arbitrary threshold was defined. It was calculated by applying a multiplication factor of 1.25 to the mean negative control signal observed in each assay run. This approach gave a fairer comparison of all the methods as different interdonor variability did not affect the value of the threshold. The value was set at 25% above the background, a level that would robustly distinguish a real change in signal response as a result of different experimental parameters to those that could be the result of method precision. Using the threshold method still allowed normalization for run-to-run variation, as the threshold value was calculated from the mean signal of the negative control samples included in each run. Only the threshold value approach was used to evaluate the different parameters for each method.

### 3.2. Sensitivity

**Figure 4:**
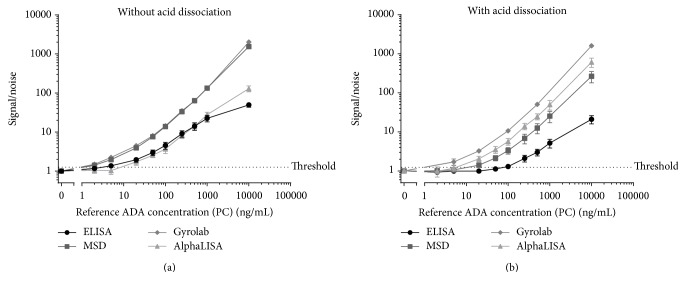
(a) PC data obtained for the interassay assessment on the different platforms without acid dissociation. The data are presented as the mean PC signal obtained divided by the mean NC signal (signal/noise). (b) PC data obtained for the interassay assessment on the different platforms with acid dissociation. The data are presented as the mean PC signal obtained divided by the mean NC signal (signal/noise).

A NC and nine PC samples were analyzed across three different days, on each of the four platforms with the two steps (with or without an acid dissociation step) (Figure 4(a)). The PC concentrations tested were 2, 5, 20, 50, 100, 250, 500, 1000, and 10000 ng/mL of reference ADA. The assay sensitivity was determined based on the results obtained across the 3 interassay runs as the lowest PC concentration consistently returning a signal above the threshold value.

All of the methods exhibited a good level of sensitivity below 100 ng/mL of reference ADA (Table 2). In the absence of an acid dissociation step, the highest sensitivities were obtained for the ELISA, Gyrolab, and MSD platforms, where the determined sensitivities were equal to 5 ng/mL, 2 ng/mL, and 2 ng/mL of reference ADA, respectively. The AlphaLISA platform also achieved a sensitivity equal to 20 ng/mL, well within target criteria, but a little lower than the other platforms. Two reasons could explain this lower sensitivity. Firstly, the final dilution factor required in all of the other technologies was 1 in 6 (1 in 3 for the target depletion and 1 in 2 for the assay) compared to 1 in 15 (1 in 3 for the target depletion and 1 in 5 for the assay) for the AlphaLISA technology (Table 2). This 1 in 5 dilutions is recommended by the provider. Secondly, the NC in the AlphaLISA assay gave a higher signal response than expected (compared to other immunoassays runs on the platform), resulting in a lower signal-to-noise ratio for samples. Further investigation work would be required such as evaluating different buffers or sample dilution parameters to address this issue. However, this would be a time-consuming step and may be viewed as a limitation of the platform in this particular assay development context.

For the methods including an acid dissociation step, the Gyrolab platform was the most sensitive (equal to 5 ng/mL of reference ADA), followed by a similar sensitivity of 20 ng/mL of reference ADA for the MSD and the AlphaLISA platforms (Table 2 and Figure 4(b)). The ELISA platform exhibited sensitivity equal to 100 ng/mL of reference ADA. The loss in sensitivity observed for most of the technologies arose from the fact that a higher dilution factor was applied when an acid dissociation step was implemented. Moreover, the dilution factor increase was not the same for all of the platforms. For the MSD and ELISA platforms, the samples were 7.5-fold more diluted (final dilution of 1 in 45 compared to 1 in 6 without acid dissociation; Table 2) whereas the final sample dilution factor was only increased from 1 in 6 to 1 in 9 for the Gyrolab technology. The Gyrolab technology defines the sample and buffer volumes within the CD microstructure, limiting the technology to a sample : acid ratio of 1 : 1. For the other 3 technologies, the sample : acid ratio was optimized as a 1 : 5 ratio, combining appropriate sensitivity, drug tolerance, and sample usage. The inability to modify the sample : acid volume ratio is seen as a small limitation of the Gyrolab assay; however, the number of samples which can be tested on each CD (48 microstructures allowing only 24 samples in duplicate) represents a bigger limitation for the use of this technology. It is noted that runs can be performed with multiple Mixing CDs (up to 5 per run), but sample numbers are still limited if appropriate controls are included on each CD and result in an increase in the assay time, though the “hands-off” benefits of automation are still retained. The only technology that showed no difference in terms of sensitivity with the acid dissociation step, despite the increased dilution factor of the samples from 1 in 15 to 1 in 45, was the AlphaLISA. This may be explained by the fact that the acid dissociation method was less sensitive to a matrix effect. Indeed for the AlphaLISA the NC signal obtained with the acid dissociation was lower than the NC signal obtained without the acid dissociation step. This suggested that the high NC signal observed without the dissociation step may have been due to the presence of a component in the matrix (serum) which was altered by the acidification process.

The dynamic range and the saturation level (hook effect) are additional parameters that can be taken into consideration for the final selection of the methods. A method exhibiting a high saturation level for anti-drug antibody concentrations is preferred. In our study, the saturation level, shown in Figures 4(a) and 4(b) for all of the methods with and without an acid dissociation step, was reached earlier on the ELISA platform in comparison to the MSD, Gyrolab, and AlphaLISA platforms. The saturation level shown for the AlphaLISA platform in the absence of an acid dissociation step is not a true representative of the assay performance due to the high background observed in the blank samples.

The sensitivity was one of the key parameters for the selection of the fit-for-purpose method but drug and target tolerance were considered of higher priority. Looking only at the sensitivity, the MSD and Gyrolab technologies displayed better results without an acid dissociation step, but the ELISA and AlphaLISA were both viable options. In the presence of the acid dissociation step, MSD and Gyrolab technologies again displayed better results. The AlphaLISA platform exhibited a similar level of performance, whilst the ELISA displayed a lower, but adequate, level of sensitivity. However, it is important to remember that the ADA assay sensitivity and saturation level are relative to the choice of the reference ADA used as a surrogate.

### 3.3. Interassay Variability

Nine different PC concentrations of 2, 5, 20, 50, 100, 250, 500, 1000, and 10000 ng/mL were tested across three different days with or without acid dissociation treatment. Due to the lower throughput on the Mixing CD (i.e., 48 sample microstructures compared to 112 microstructures on a Bioaffy CD200), only five concentrations of PCs were tested per run on Gyros. Instead, the five PC concentrations of 5, 20, 100, 500, and 10000 ng/mL were tested across 5 different runs. The precision (CV%) obtained for the different runs performed for each method was calculated on the signal value obtained at each PC level and the results are presented in Table 3.

For this stage of development, all of the methods demonstrated an acceptable interassay precision. For the eight methods, the PCs with a concentration above the threshold achieved a precision below 27% (Table 3). However, differences were observed between the different methods. Without acid dissociation, the better interassay precision was obtained on the Gyrolab and MSD platforms with a precision below 10% for all of the PC samples. For the two other platforms (ELISA and AlphaLISA), precision between 4% and 27% was obtained for the methods without an acid dissociation step. For ELISA and AlphaLISA platforms, the precisions were similar (below 19%) with the addition of an acid dissociation step. For the MSD platform, an increase in imprecision from 10% to 26% was clearly observed with the method containing an acid dissociation step. For the Gyrolab method only the 5 ng/mL PC sample produced a CV greater than 10%; this was almost certainly due to the automation of the Gyrolab platform that reduced the manual handling work and thus the variability. As this work corresponds to a preliminary assay evaluation, the precision obtained with the acid dissociation step might be improved in the future with a refinement of the method.

The interassay precision was assessed only on samples containing free ADA with and without an acid dissociation treatment. It was not tested whether the presence of Novimab in the samples and the target depletion step would affect the interassay precision. There is indeed a risk that the additional treatment steps might increase the interassay variability. Whilst this has not been assessed for all the methods, interdonor variability has been assessed for the MSD method with the inclusion of target depletion and acid dissociation steps and resulted in no noticeable increase in the precision.

### 3.4. Interdonor Variability

**Figure 5:**
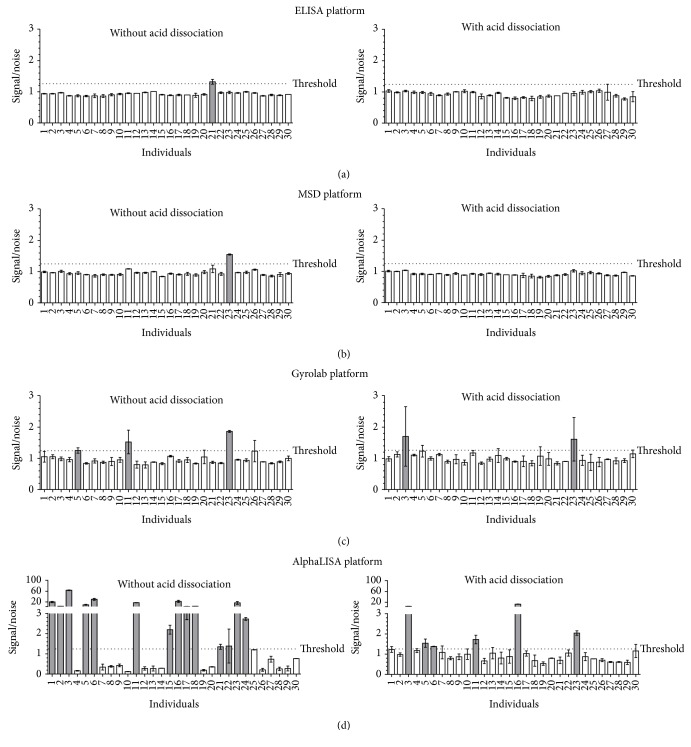
Interdonor variability according to the platform used and the treatment applied to the samples. The signals obtained for the 30 donors were normalized in signal/noise values where the noise represents the mean NC signal measured in each method. The dotted line represents the threshold fixed at a signal/noise value equal to 1.25. The individual sera giving a signal/noise above the threshold were identified and the corresponding bars in the graphs were colored grey.

Thirty naïve individual sera were tested to assess the interdonor variability on the different platforms with or without an acid dissociation step. The results are shown in Figure 5.

During this study, thirty naïve individual sera were tested in order to establish a RSCP and to evaluate the interdonor variability. As explained previously in Analysis Strategy, the approach based on a statistical cut-point was not appropriate in this study. Consequently, only the interdonor variability is presented in Figure 5. For all of the methods except for the AlphaLISA, the signal response of the serum matrix pool used for the negative controls and preparation of the positive controls was representative of the signal response of the individual matrices. Similar signals were obtained for the blanks and for the 30 naïve individual sera. Consequently, the second approach for the calculation of the cut-point based on a threshold was used to determine the number of donors that would be considered as false positives. For the AlphaLISA platform using a method without an acid dissociation step, a high background was observed with the serum matrix pool. As previously explained, more investigations would be required to understand the origin of this high background.

A very low interdonor variability was observed for the ELISA and MSD platforms in the presence or absence of an acid dissociation step. For both technologies, a maximum of one individual, which represents less than 5% of the donors, exhibited a signal above the threshold. A higher variability was observed with the Gyrolab platform. Indeed, 3 and 2 naïve individuals out of the 30 naïve individuals tested produced a signal above the threshold, for the method without or with acid dissociation, respectively. In some cases, it was due to an outlier in the duplicate data leading to a sample precision greater than 30%. This phenomenon is occasionally observed with this technology. As it is an instrument based on microfluidics the matrix should be free of particulate matter that could interfere in the assay and result in high signal for one of the replicates. The AlphaLISA platform showed a very high interdonor variability, especially without the acid dissociation step where almost 50% (14 out 30) of the naïve individuals were above the threshold and considered as false positives. The results were improved with the acid dissociation step with 20% (6 out 30) of the individuals above the threshold but the data still remained out of criteria.

As a conclusion, the ELISA, MSD, and Gyrolab platforms displayed an acceptable interdonor variability. No selectivity issues would be anticipated for assay validation on the ELISA and MSD platforms. For the Gyrolab platform, additional care may be required with sample handling to avoid outliers and thus this can be seen as a limitation of this platform. For the AlphaLISA technology, investigation work would be required such as evaluating different buffers or sample dilution parameters to address this issue. However, this would represent a time-consuming step and therefore may be viewed as a limitation of the platform in this particular assay development context.

### 3.5. Drug Tolerance

Based on our previous clinical data, Novimab levels approaching 20 *μ*g/mL were anticipated in clinical samples during the ADA assessment. As previously described this can lead to false negative results during the clinical sample testing phase if the assay does not have sufficient tolerance. Acid dissociation is a well-documented approach that can be used to overcome drug interference problems. In order to evaluate the impact of an acid dissociation step, the comparison study was performed on the 4 platforms, with and without an acid dissociation step. The acid dissociation step was optimized on each platform and the optimal conditions were used. The results are presented in Table 4.

Nine PC samples (5, 20, 50, 100, 250, 500, 1000, 2500, and 10000 ng/mL) were analyzed in the presence of 0, 20, and 100 *μ*g/mL concentrations of Novimab. Table 4 shows the drug tolerance data obtained for two concentrations of Novimab (20 and 100 *μ*g/mL) for both treatments tested on the 4 different platforms.

In the absence of an acid treatment step, the Gyrolab platform showed the highest level of drug tolerance where 100 ng/mL and 500 ng/mL of reference ADA could be detected in the presence of 20 *μ*g/mL and 100 *μ*g/mL of Novimab, respectively. The MSD platform also performed well because 250 ng/mL and 1000 ng/mL of reference ADA could be detected in presence of 20 *μ*g/mL and 100 *μ*g/mL of Novimab, respectively. The ELISA and the AlphaLISA assays showed a critical loss of sensitivity in the presence of Novimab when no acid dissociation was performed.

Despite the decrease in sensitivity for the acidified samples containing no Novimab, except for the AlphaLISA where it is equivalent with and without acid dissociation, the drug tolerance level was generally improved when an acid treatment was performed, consistent with previous publications [6–10, 13]. The acid treatment step dissociates the ADA-therapeutic drug complexes, thus increasing the accessibility of the capture and detection reagents to the ADA. However, whilst the drug tolerance was improved by the acid treatment on all of the platforms, the performances still varied across them. Thus when considering the 20 *μ*g/mL Novimab level, the Gyrolab platform exhibited a higher level of drug tolerance than the MSD, the ELISA, and the AlphaLISA, as these platforms allowed detection of 5, 50, 100, and 1000 ng/mL of reference ADA, respectively. With 100 *μ*g/mL of Novimab, the Gyrolab and the MSD platforms still exhibited the highest level of drug tolerance because 50 ng/mL of reference ADA could be detected.

Taken together, these data suggest that the acid dissociation treatment is efficient in increasing the drug tolerance level by a factor 5- to 40-fold. Despite this increase some platforms still exhibited a poor level of drug tolerance (e.g., AlphaLISA). In this particular study, the Gyrolab and the MSD were the two technologies where a sensitivity of 50 ng/mL of reference ADA could be achieved in the presence of 20 *μ*g/mL of Novimab, when an acid dissociation step was performed.

### 3.6. Target Tolerance

A major challenge encountered in immunogenicity assays is the level of target tolerance. The presence of dimeric or multimeric forms of a soluble target may induce a signal in a bridging immunogenicity assay and lead to a false positive result. In our example, the soluble target has the capacity to form dimers that can interfere in the assay.

In some disease populations, the basal level of soluble dimeric target can be considerably higher than the level measured in a healthy population. It is also known that injection of an antibody directed against a soluble dimeric target can lead to the accumulation of the latter in the serum due to the different clearance rate of the therapeutic drug-soluble target complex [21]. Based on the anticipated soluble dimeric target level in the disease population and its expected accumulation during the treatment period, it was important to improve the target tolerance of our assay format.

Methods to improve the target tolerance can include addition of a high concentration of a competitive anti-target antibody to the samples to compete with the labeled drugs for the binding to the target. This approach was tested with several competing anti-target antibodies and whilst the target interference was improved even a 300-fold molar excess of competing anti-target antibody compared to the target was not enough to reduce the interference of the target below the threshold of the assay, leading to the generation of false positive results (data not shown). Consequently, immunodepletion of the target was implemented as described in Section 2. This is important to keep in mind that such additional sample processing should be applied only if needed because it may cause a loss of the low affinity ADA (e.g., IgM) due to the additional processing step. The impact of sample processing on detection of low affinity ADA was not investigated in the context of this work.

In the comparison study, in order to evaluate the efficiency of the target depletion procedure, samples containing increasing concentrations of soluble dimeric target were split into 2 groups, with and without a target depletion step. The samples not depleted were diluted in PBS-1% BSA 0.05% tween to mimic the depletion step dilution and to maintain the appropriate final dilution. To identify the optimal method to enhance the target tolerance in the ADA assay, samples containing four different concentrations of soluble dimeric target (5, 20, 50, and 200 ng/mL) were analyzed across the different technologies. The samples that had undergone different treatments, that is, with/without acid dissociation and with/without target depletion, were finally compared.

**Figure 6:**
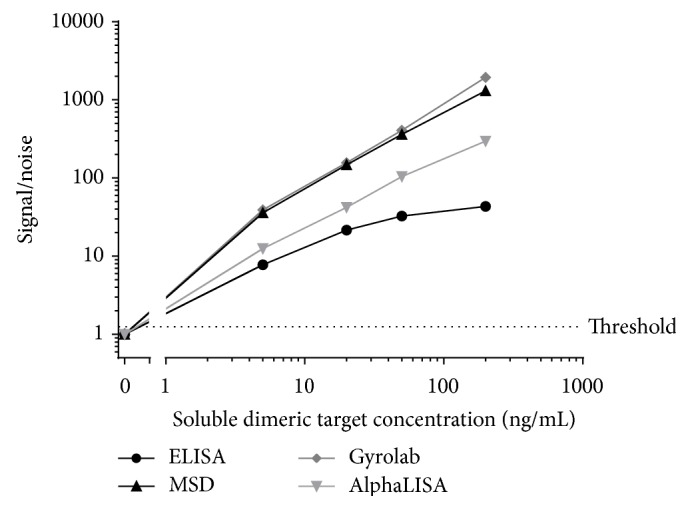
Evaluation of target interference for each assay in the presence of 0, 5, 20, 50, and 200 ng/mL of soluble dimeric target evaluated in the method without acid dissociation or target depletion. The noise represents the mean NC signal measured in each method. The dotted line represents the threshold fixed at a signal/noise value equal to 1.25.

With no acid dissociation and no target depletion, even a low level of soluble dimeric target of 5 ng/mL generated a false positive signal on all of the platforms (Table 5 and Figure 6). The soluble dimeric target interfering in the assay is acid-labile and the addition of an acid dissociation treatment step alone was sufficient to achieve the desired target tolerance of 50 ng/mL in the ELISA assay. This was a clear advantage compared to the other technologies where further sample processing in the form of a target depletion step was required to reach the required target tolerance level. However, the ELISA assay with acid treatment did have lower albeit adequate sensitivity.

Indeed across all of the platforms and with both methods (i.e., with or without acid dissociation), the target depletion procedure enhanced the levels of target tolerance (Table 5). However, in the absence of acid dissociation, the MSD, the Gyrolab, and the AlphaLISA were only tolerant to 5 ng/mL of soluble dimeric target with target depletion alone. This level of target could be expected in clinical samples and thus remained problematic. The ELISA displayed the highest level of target tolerance, up to 20 ng/mL, with target depletion alone (Table 5) and also with acid dissociation alone, thus showing clear advantages for the target tolerance evaluation parameter.

For all of the 4 technologies, the highest level of target tolerance was obtained with a combination of the target depletion and acid dissociation steps. With these combined sample treatments the target tolerance was increased to 200 ng/mL of soluble dimeric target for all of the platforms assessed.

## 4. Conclusion

The aim of this study was to develop an ADA assay on different technology platforms (ELISA, MSD, Gyrolab, and AlphaLISA) with different additional steps (±acid dissociation, ±target depletion) in order to achieve an assay with an appropriate sensitivity, interdonor variability, drug, and target tolerance level.

All of the 4 platforms were tested either with or without acid dissociation and with or without target depletion. The data obtained in this study showed that the acid dissociation step improved the drug tolerance by a factor of 5 to 40 depending on the technology used and the drug concentration. For the expected Novimab concentration in the clinical samples, that is, 20 *μ*g/mL, the Gyrolab and MSD platforms showed a drug tolerance level equal to 5 ng/mL and 50 ng/mL of reference ADA, respectively. These values satisfied our 50 ng/mL sensitivity criteria for the reference ADA. The drug tolerance for the ELISA and AlphaLISA platforms was also improved by the acid dissociation treatment but the level remained below our target criteria (100 ng/mL and 1000 ng/mL of reference ADA in presence of 20 *μ*g/mL of Novimab, resp.). The acid dissociation treatment also had a positive impact on the target tolerance as the soluble dimeric target is acid-labile. For a soluble dimeric target concentration of 50 ng/mL this treatment alone was sufficient to lower the signal below the threshold value for the ELISA platform. This is a clear advantage for the ELISA platform where less sample processing was required to meet our requirements in terms of drug and target tolerance, whilst achieving adequate sensitivity. For the MSD technology, inclusion of an acid dissociation step improved target tolerance a little; for Gyrolab and AlphaLISA no marked improvement was noted. For these latter 3 technologies the signal remained above the threshold value at the required target tolerance level of 50 ng/mL, thus producing false positive results. This interference was overcome by combining a target depletion step with the acid treatment. For all platforms this was efficient to raise the target tolerance to 200 ng/mL of soluble dimeric target. Despite all of the advantages in terms of drug and target tolerance obtained by the inclusion of acid treatment, it did have a negative impact on sensitivity and the interassay variability parameters. The sensitivity was indeed reduced due to a higher sample dilution required by the acid treatment. The increase in interassay variability observed with the acid dissociation treatment is likely a result of the additional sample processing steps which had to be performed during the assay. However, despite this these two parameters still remained within our acceptance criteria.

There are a number of different technologies available for the development of ADA assays. In this study the ELISA, Gyrolab, MSD, and AlphaLISA were evaluated. The MSD, the ELISA, and the Gyrolab appeared to be the 3 platforms that gave the better results in terms of sensitivity, interassay precision, interdonor variability, drug tolerance, and target tolerance. Each platform has particular advantages. The ELISA platform exhibited a relative sensitivity slightly lower than MSD and Gyrolab technology and when acid dissociation treatment was applied, the sensitivity was reduced to 100 ng/mL, less than our target criteria, although adequate in terms of industry expectations. In achieving our requirements in terms of drug and target tolerance, with less sample pretreatment steps, the ELISA represents an attractive option. Additionally, from a practical perspective, implementation of this methodology in the laboratory environment requires no specialist immunoassay platforms. However, a disadvantage of the ELISA was that the signal response of the NC was often close to the lower limit of detection of the reader with a consequent decreased interdonor variability which could be problematic for the selectivity and cut-point assessments during the assay validation phase. This parameter could probably be improved by optimizing the incubation times, reagent concentrations, or sample dilutions to slightly increase the background signal.

With the MSD and the Gyrolab technologies, for the key parameters of drug tolerance and target tolerance, the best performance was obtained when the samples were subjected to both acid dissociation and target depletion procedures. In this case the interassay variability was higher when using the MSD platform compared to the Gyrolab. This almost certainly comes from the automation of this latter technology which provides a better reproducibility between each run. Additionally, the sample volume requirements are lower for the Gyrolab than the MSD. However, the Gyrolab requires a more regular and time-consuming maintenance than the MSD reader and is subject to more technical challenges. For the present ADA assay application the throughput is limited by the availability of only 48 microstructures on the Gyrolab Mixing CD used to perform the acid dissociation treatment. Specific care is required during the sample handling and assay preparation to avoid the presence of outliers. The matrix should be free of particulate matter that could block the microstructures on the CDs and the stickiness of the Alexa Fluor conjugated detection antibodies requires specific handling to avoid aggregate formation, both of which can result in anomalous fluorescence peaks.

The AlphaLISA platform cannot be considered a suitable technology for further development of this particular assay because a lower drug tolerance and a higher interdonor variability were observed compared to the other platforms. Other technology comparison studies have reached different conclusions concerning their preferred choice of technology [18]; however, as demonstrated in this study, it is important to consider the individual requirements of each particular clinical study to select the most appropriate assay and platform. Factors that also need to be considered in addition to assay performance are available sample volume, assay run time, automation, reagent costs, and platform availability at Contract Research Organizations (CROs). Therefore, the testing of multiple technology platforms, in parallel, during method development, is recommended to facilitate the selection of the most appropriate ADA screening assay format to support a clinical trial program.

## Figures and Tables

**Table 1:** Sample dilution factors according to the different methods (platforms and additional steps).

Technology used	ELISA				MSD				Gyrolab				AlphaLISA			
Acid dissociation step	No		Yes		No		Yes		No		Yes		No		Yes	
Target depletion step	No	Yes	No	Yes	No	Yes	No	Yes	No	Yes	No	Yes	No	Yes	No	Yes
Dilution induced by the target depletion step^(1)^	1 in 3		1 in 3		1 in 3		1 in 3		1 in 3		1 in 3		1 in 3		1 in 3	
Dilution induced by the acidification step	NA		1 in 5		NA		1 in 5		NA		1 in 2		NA		1 in 5	
Dilution induced by the neutralization step/Master Mix addition	1 in 2		1 in 3		1 in 2		1 in 3		1 in 2		1 in 1.5		1 in 5		1 in 3	
*Final dilution applied before platform analysis*	*1 in 6*		*1 in 45*		*1 in 6*		*1 in 45*		*1 in 6*		*1 in 9*		*1 in 15*		*1 in 45*	

^(1)^Samples not target depleted were diluted at 1 in 3 in PBS-1% BSA 0.05% tween.

**Table 2:** Sensitivity of the different methods determined as the lowest PC concentration exhibiting a response above the threshold.

Technology used	ELISA				MSD				Gyrolab				AlphaLISA			
Acid dissociation step	No		Yes		No		Yes		No		Yes		No		Yes	
Target depletion step	No	Yes	No	Yes	No	Yes	No	Yes	No	Yes	No	Yes	No	Yes	No	Yes
Final dilution applied before platform analysis	1 in 6		1 in 45		1 in 6		1 in 45		1 in 6		1 in 9		1 in 15		1 in 45	
Sensitivity ng/mL of reference ADA	5		100		2		20		2		5		20		20	

**Table 3:** Interassay precision obtained on the different methods for nine levels of PCs (five levels of PCs were assessed on the Gyrolab platform with acid dissociation due to the lower number of structures present on the Gyrolab Mixing CDs). The precision is presented in %CV and is calculated from three independent runs performed on different days (except for the Gyrolab assay with acid dissociation where five independent runs were performed). Each run contained two sets of independently prepared PCs.

	Interassay precision (CV%)							
Technology used	ELISA		MSD		Gyrolab		AlphaLISA	
Acid dissociation step	No	Yes	No	Yes	No	Yes	No	Yes
Reference ADA concentration (PC) in ng/mL								
2	*<BLQ*	*<BLQ*	5.51	*<BLQ*	8.39	NT	*<BLQ*	*<BLQ*
5	14.83	*<BLQ*	6.00	*<BLQ*	6.03	16.07	*<BLQ*	*<BLQ*
20	20.14	*<BLQ*	4.99	4.03	1.67	3.88	14.49	17.11
50	21.94	*<BLQ*	5.45	8.96	3.11	NT	9.46	14.43
100	24.67	18.89	3.09	11.57	2.58	5.04	10.34	13.83
250	23.81	5.66	4.62	21.46	4.97	NT	12.91	14.67
500	26.77	7.30	5.31	22.47	3.61	6.07	16.23	9.37
1000	23.56	2.50	4.73	25.98	2.89	NT	11.44	18.99
10000	13.41	1.73	6.37	25.95	6.87	7.21	4.19	4.76

“NT” was used for the PCs not tested due to the limited number of structures on Gyrolab Mixing CDs. “<BLQ” refers to a result below the sensitivity of the method.

**Table 4:** Sensitivity observed for each assay in the presence of 0, 20, and 100 *µ*g/mL of Novimab. The sensitivity was calculated as the lowest PC concentration where the signal obtained was above the threshold value.

	Sensitivity (ng/mL of reference ADA)							
Technology used	ELISA		MSD		Gyrolab		AlphaLISA	
Acid dissociation step	No	Yes	No	Yes	No	Yes	No	Yes
Novimab concentration (*μ*g/mL)								
0	5	100	2	20	2	5	20	20
20	1000	100	250	50	100	5	10000	1000
100	10000	250	1000	50	500	50	>10000	10000

**Table 5:** Results obtained on the different methods according to the levels of soluble dimeric target.

Technology used	ELISA				MSD				Gyrolab				AlphaLISA			
Acid dissociation step	No		Yes		No		Yes		No		Yes		No		Yes	
Depleted samples	No	Yes	No	Yes	No	Yes	No	Yes	No	Yes	No	Yes	No	Yes	No	Yes
Soluble Target Concentration (ng/mL)																
5	+	−	−	−	+	−	−	−	+	−	+	−	+	−	+	−
20	+	−	−	−	+	+	+	−	+	+	+	−	+	+	+	−
50	+	+	−	−	+	+	+	−	+	+	+	−	+	+	+	−
200	+	+	+	−	+	+	+	−	+	+	+	−	+	+	+	−

The symbols “+” and “−” refer to sample exhibiting a signal above or below the threshold value, respectively.

## Abbreviations

ACP: Assay cut-point
ADA: Anti-drug antibody
AF: Alexa Fluor 647
AlphaLISA: Amplified luminescent proximity homogeneous assay developed by PerkinElmer Company
BLQ: Below level of quantification
CRO: Contract Research Organization
ELISA: Enzyme-linked immunosorbent assay
FDA: Food and Drug Administration
Gyrolab: Automated microfluidics immunoassay developed by Gyros AB
HRP: Horseradish Peroxidase
MSD: Electrochemiluminescent immunoassay developed by Meso Scale Discovery
NC: Negative control
NF: Normalization factor
Novimab: Fully human therapeutic monoclonal antibody
NT: Not tested
PC: Positive control reference ADA
RPM: Rotations per minute
RSCP: Run specific cut-point
sTAG: SULFO-TAG
Th: Threshold.
